# Positive periodic solution for *p*-Laplacian neutral Rayleigh equation with singularity of attractive type

**DOI:** 10.1186/s13660-018-1654-6

**Published:** 2018-03-14

**Authors:** Yun Xin, Hongmin Liu, Zhibo Cheng

**Affiliations:** 1 College of Computer Science and Technology, Henan Polytechnic University, Jiaozuo, China; 2 School of Mathematics and Information Science, Henan Polytechnic University, Jiaozuo, China; 3 Department of Mathematics, Sichuan University, Chengdu, China

**Keywords:** Neutral operator, *p*-Laplacian, Periodic solution, Rayleigh equation, Singularity of attractive type

## Abstract

In this paper, we consider a kind of *p*-Laplacian neutral Rayleigh equation with singularity of attractive type,

$$\bigl(\phi_{p} \bigl(u(t)-cu(t-\delta) \bigr)' \bigr)'+f \bigl(t,u'(t) \bigr)+g \bigl(t, u(t) \bigr)=e(t). $$

By applications of an extension of Mawhin’s continuation theorem, sufficient conditions for the existence of periodic solution are established.

## Introduction

As is well known, the Rayleigh equation can be derived from many fields, such as physics, mechanics and engineering technique fields, and an important question is whether this equation can support periodic solutions. In 1977, Gaines and Mawhin [1] introduced some continuation theorems and applied this theorem to discussing the existence of solutions for the Rayleigh equation [1, p. 99]

$$u''+f \bigl(u' \bigr)+g(t,u)=0. $$

Gaines and Mawhin’s work has attracted the attention of many scholars in the field of the Rayleigh equations. More recently, the existence of periodic solutions for Rayleigh equation was extensively studied (see [2–11] and the references therein). Some classical tools have been used to study Rayleigh equation in the literature, including the method of upper and lower solutions [6], the time map continuation theorem [7, 9], fixed point theory [4], the Manásevich–Mawhin continuation theorem [10, 11], and coincidence degree theory [2, 3, 5, 8].

Recently there have been published some results on singular Rayleigh equations [12–16]. In 2015, Wang and Ma [15] investigated the following singular Rayleigh equation:

$$u''+f \bigl(t,u' \bigr)+g(u)=p(t), $$

where *g* had a repulsive singularity at the origin, i.e.,

1.1 $$ \lim_{u\to 0^{+}} g(u)=-\infty. $$

By applications of the limit properties of the time map, the authors obtained the result of the existence of periodic solution for this equation. Afterwards, by using topological degree theory, Chen and Lu [12] discussed that the existence of periodic solution for the following singular Rayleigh equations:

1.2 $$ u''+f \bigl(t,u' \bigr)+ \varphi (t)u(t)-\frac{1}{u^{r}(t)}=h(t). $$

The authors found new methods for estimating a lower priori bounds of periodic solutions to equation (1.2). Recently, Xin and Cheng [16] investigated a kind of neutral Rayleigh equation with singularity of repulsive type,

1.3 $$ \bigl(u(t)-cu(t-\delta) \bigr)''+f \bigl(t,u'(t) \bigr)+g \bigl(t,u(t) \bigr)=e(t), $$

where $g(t,u)=g_{1}(t,u)+g_{0}(u)$ and $g_{0}$ had a strong singularity at $u=0$, i.e.,

1.4 $$ \lim_{u\to 0^{+}} \int^{u}_{1}g_{0}(s)\,ds=+\infty. $$

By applications of coincidence degree theory, the authors found the existence of positive periodic solution for equation (1.3).

All the aforementioned results are related to Rayleigh equation or neutral Rayleigh equation with singularity of repulsive type. Naturally, a new question arises: how *p*-Laplacian neutral Rayleigh equation works on singularity of attractive type? Besides practical interests, the topic has obvious intrinsic theoretical significance. To answer this question, in this paper, we consider a kind of *p*-Laplacian neutral Rayleigh equation with singularity of attractive type,

1.5 $$ \bigl(\phi_{p} \bigl(u(t)-cu(t-\delta) \bigr)' \bigr)'+f \bigl(t,u'(t) \bigr)+g \bigl(t, u(t) \bigr)=e(t), $$

where $p>1$, $\varphi_{p}(u)=\vert u\vert ^{p-2}u$ for $u\neq 0$ and $\varphi _{p}(0)=0$; $\vert c\vert \neq 1$ and *δ* is a constant with $0\leq \delta <\omega $; $e:\mathbb{R}\rightarrow \mathbb{R}$ is continuous periodic functions with $e(t+\omega)-e(t)\equiv 0$ and $\int^{T}_{0}e(t)\,dt=0$; *f* is for continuous functions defined on $\mathbb{R}^{2}$ and periodic in *t* with $f(t,\cdot)=f(t+\omega, \cdot)$ and $f(t,0)=0$, $g(t,u)=g_{0}(u)+g_{1}(t,u)$, here $g_{1}:\mathbb{R}\times (0,+\infty)\to \mathbb{R}$ is an $L^{2}$-Carathéodory function, $g_{1}(t,\cdot)=g_{1}(t+\omega, \cdot)$; $g_{0}\in C((0,\infty);\mathbb{R})$ has an attractive singularity at the origin, i.e.,

1.6 $$ \lim_{u\to 0^{+}} \int^{u}_{1}g_{0}(s)\,ds=-\infty. $$

Obviously, the attractive condition (1.6) is in contradiction with the repulsive singularity of (1.1) and (1.4). Therefore, the above methods of [12, 15, 16] are no long applicable to the proof of existence of a periodic solution for (1.5) with singularity of attractive type. So we need to find a new method to get over it.

In this paper, by applications of an extension of Mawhin’s continuation theorem in [17] and some analysis techniques, we see the existence of a positive periodic solution for (1.5). Our results improve and extend the results in [12, 15, 16].

## Preliminary lemmas

For convenience, define

$$C^{1}_{\omega }= \bigl\{ u\in C^{1}(\mathbb{R}, \mathbb{R}): u(t+\omega)=u(t) \bigr\} , $$

which is a Banach space endowed with the norm $\Vert \cdot \Vert $ define by $\Vert u\Vert =\max \{\Vert u\Vert _{\infty },\Vert u'\Vert _{\infty }\}$, for all *x*, and

$$\Vert u\Vert _{\infty }=\max_{t\in [0,\omega ]} \bigl\vert u(t) \bigr\vert ,\qquad \bigl\Vert u' \bigr\Vert _{\infty }= \max_{t\in [0,\omega ]} \bigl\vert u'(t) \bigr\vert . $$

### Lemma 2.1

(see [18])

*If* $\vert c\vert \neq 1$, *then the operator* $(Au)(t):=u(t)-cu(t- \delta)$ *has a continuous inverse* $A^{-1}$ *on the space* $C_{\omega }$, *and satisfying*

$$\bigl( A^{-1}f\bigr) (t)= \textstyle\begin{cases} f(t)+ \sum^{\infty }_{j=1}c^{j}f(t-j\delta), & \textit{for } \vert c\vert < 1, \forall f\in C_{\omega }, \\ -\frac{f(t+\delta)}{c}-\sum^{\infty }_{j=1}\frac{1}{c^{j+1}}f(t+(j+1) \delta),& \textit{for } \vert c\vert >1, \forall f\in C_{\omega }. \end{cases} $$

### Lemma 2.2

*If* $\vert c\vert \neq 1$, *then operator* $A^{-1}$ *satisfying*

$$\int^{\omega }_{0}\bigl\vert \bigl( A^{-1}f \bigr) (t)\bigr\vert ^{p}\,dt\leq \frac{1}{\vert 1-\vert c\vert \vert ^{p}} \int^{\omega }_{0}\bigl\vert f(t)\bigr\vert ^{p}\,dt, \quad \forall f \in C_{\omega }, \textit{here } 1\leq p< \infty. $$

### Proof

We first consider $\vert c\vert <1$. From Lemma 2.1, we have

$$\begin{aligned} \int^{\omega }_{0} \bigl\vert \bigl( A^{-1}f \bigr) (t) \bigr\vert ^{p}\,dt = & \int^{\omega }_{0} \Biggl\vert \sum _{j=0}^{\infty }c^{j} f(t-j\delta) \Biggr\vert ^{p}\,dt \\ \leq & \int^{\omega }_{0} \Biggl( \sum _{j=0}^{\infty } \bigl\vert c^{j}f(t-j\delta) \bigr\vert \Biggr) ^{p}\,dt \\ \leq &\frac{1}{(1-\vert c\vert )^{p}} \int^{\omega }_{0} \bigl\vert f(t) \bigr\vert ^{p}\,dt. \end{aligned}$$

Similarly, for $\vert c\vert >1$, we can get

$$\int^{\omega }_{0} \bigl\vert \bigl( A^{-1}f \bigr) (t) \bigr\vert ^{p}\,dt\leq \frac{1}{(\vert c\vert -1)^{p}} \int^{\omega }_{0} \bigl\vert f(t) \bigr\vert ^{p}\,dt. $$

Therefore, we have

$$\int^{T}_{0} \bigl\vert \bigl( A^{-1}f \bigr) (t) \bigr\vert ^{p}\,dt\leq \frac{1}{\vert 1-\vert c\vert \vert ^{p}} \int^{T}_{0} \bigl\vert f(t) \bigr\vert ^{p}\,dt. $$

 □

### Lemma 2.3

(see [19])

*If* $u\in C^{1}_{\omega }(\mathbb{R},\mathbb{R})$, *and there exists a point* $t^{*}\in [0,\omega ]$ *such that* $\vert u(t^{*})\vert < d$, *then*

$$\Vert u\Vert _{\infty }\leq d+\frac{1}{2} \int^{\omega }_{0} \bigl\vert u(t) \bigr\vert '\,dt $$

*and*

$$\biggl( \int^{\omega }_{0} \bigl\vert u(t) \bigr\vert ^{p}\,dt \biggr) ^{\frac{1}{p}}\leq \biggl( \frac{ \omega }{\pi_{p}} \biggr) \biggl( \int^{\omega }_{0} \bigl\vert u'(t) \bigr\vert ^{2}\,dt \biggr) ^{\frac{1}{p}}+d\omega^{\frac{1}{p}}, $$

*where* $1\leq p<\infty $, $\pi_{p}=2\int^{(p-1)/p}_{0}\frac{ds}{(1-\frac{s ^{p}}{p-1})^{1/p}}=\frac{2\pi (p-1)^{1/p}}{p\sin (\pi /p)}$.

The following lemma involves the consequences of Theorem 3.1 of [17].

### Lemma 2.4

*Assume that condition* $\vert c\vert \neq 1$, Ω *is an open bounded set in* $C^{1}_{\omega }$. *If*:

(i) *for each* $\lambda \in (0,1)$ *the equation*
  2.1 $$ \bigl(\phi_{p}(Au)'(t) \bigr)'+\lambda f \bigl(t,u'(t) \bigr)+\lambda g \bigl(t,u(t) \bigr)=\lambda e(t) $$
  *has no solution on* *∂*Ω;
(ii) *the equation*
  $$F(a):=\frac{1}{\omega } \int^{\omega }_{0}g(t,a)\,dt=0 $$
  *has no solution on* $\partial \Omega \cap \mathbb{R}$;
(iii) *the Brouwer degree*
  $$\deg \{F,\Omega \cap \mathbb{R},0\}\neq 0, $$
  *then Eq*. (2.1) *has at least one periodic solution on* Ω̄.

## Main results: positive periodic solution for (1.5)

In this section, we will consider the existence of a positive periodic solution for (1.5) with singularity.

### Theorem 3.1

*Assume that the following conditions hold*:

$(H_{1})$
*there exists a positive constant* *K* *such that* $\vert f(t,v)\vert \leq K$, *for* $(t,v)\in \mathbb{R}\times \mathbb{R}$;
$(H_{2})$
*there exist positive constants* $D_{1}$ *and* $D_{2}$ *with* $D_{1}>D_{2}>0$ *such that* $g(t,u)<-K$ *for* $(t,u)\in \mathbb{R}\times (D_{1},+\infty)$ *and* $g(t,u)>K$ *for* $(t,u)\in \mathbb{R}\times (0,D _{2})$;
$(H_{3})$
*there exist positive constants* *a*, *b* *such that*
  $$-g(t,u)\leq a u^{p-1}+b,\quad \textit{for all } u>0. $$

*Then* (1.5) *has at least one positive solution with period* *ω* *if* $\frac{\omega (1+\vert c\vert )^{\frac{1}{p}}a^{\frac{1}{p}}}{\vert 1-\vert c\vert \vert }<2^{ \frac{p-1}{p}}$.

### Proof

Firstly, we will claim that the set of all possible *ω*-periodic solutions of (2.1) is bounded. Let $u(t)\in C^{1}_{\omega }$ be an arbitrary solution of (2.1) with period *ω*.

We claim that there exists a point $t_{0}\in [0,\omega ]$ such that

3.1 $$ 0< u(t_{0})\leq D_{1}. $$

Integrating both sides of (2.1) over $[0,\omega ]$, we have

3.2 $$ \int^{\omega }_{0} \bigl[f \bigl(t,u'(t) \bigr)+g \bigl(t,u(t) \bigr) \bigr]\,dt=0. $$

Therefore, from $(H_{1})$, we have

$$-K\omega \leq \int^{\omega }_{0}g \bigl(t,u(t) \bigr)\,dt\leq K\omega. $$

From $(H_{2})$, we know that there exist two points $t_{0}$, $\tau \in (0,T)$, such that

3.3 $$ u(t_{0})\leq D_{1}, \quad \mbox{and}\quad u( \tau)>D_{2}. $$

Since $u(t)>0$, $t\in [0,\omega ]$, we get $0< u(t_{0})\leq D_{1}$. Equation (3.1) is proved.

Then, from Lemma 2.3, we have

3.4 $$ \begin{aligned} \Vert u\Vert _{\infty } & \leq D_{1}+\frac{1}{2} \int^{\omega }_{0} \bigl\vert u'(s) \bigr\vert \,ds. \end{aligned} $$

Multiplying both sides of (2.1) by $(Au)(t)$ and integrating over $[0,\omega ]$, we get

$$\begin{aligned}& \int^{\omega }_{0} \bigl(\phi_{p}(Au)'(t) \bigr)'(Au) (t)\,dt+\lambda \int^{\omega } _{0}f \bigl(t,u'(t) \bigr) (Au) (t)\,dt+\lambda \int^{\omega }_{0}g \bigl(t,u(t) \bigr) (Au) (t)\,dt \\& \quad = \lambda \int^{\omega }_{0} e(t) (Au) (t)\,dt, \end{aligned}$$

i.e.

3.5 $$\begin{aligned} \int^{\omega }_{0} \bigl\vert (Au)'(t) \bigr\vert ^{p}\,dt =&\lambda \int^{\omega }_{0}f \bigl(t,u'(t) \bigr) (Au) (t)\,dt+ \lambda \int^{\omega }_{0}g \bigl(t,u(t) \bigr) (Au) (t)\,dt \\ &{}- \lambda \int^{\omega } _{0} e(t) (Au) (t)\,dt. \end{aligned}$$

From $(H_{1})$, we have

3.6 $$\begin{aligned}& \int^{\omega }_{0} \bigl\vert (Au)'(t) \bigr\vert ^{p}\,dt \\& \quad \leq \bigl(1+\vert c\vert \bigr) \int^{\omega }_{0} \bigl\vert f \bigl(t,u'(t) \bigr) \bigr\vert \bigl\vert u(t) \bigr\vert \,dt+ \int^{\omega }_{0} \bigl\vert g \bigl(t,u(t) \bigr) \bigr\vert \bigl\vert u(t) \bigr\vert \,dt+ \int^{\omega }_{0} \bigl\vert e(t) \bigr\vert \bigl\vert u(t) \bigr\vert \,dt \\& \quad \leq \bigl(1+\vert c\vert \bigr)\Vert u\Vert _{\infty } \biggl( \int^{\omega }_{0} \bigl\vert f \bigl(t,u'(t) \bigr) \bigr\vert \,dt+ \int^{\omega }_{0} \bigl\vert g \bigl(t,u(t) \bigr) \bigr\vert \,dt + \int^{\omega }_{0} \bigl\vert e(t) \bigr\vert \,dt \biggr) \\& \quad \leq \bigl(1+\vert c\vert \bigr)\Vert u\Vert _{\infty } \biggl( K \omega +\Vert e\Vert _{\infty }\omega + \int^{\omega }_{0} \bigl\vert g \bigl(t,u(t) \bigr) \bigr\vert \,dt \biggr). \end{aligned}$$

We get from $(H_{1})$, $(H_{3})$ and (3.2)

3.7 $$\begin{aligned} \int^{\omega }_{0} \bigl\vert g \bigl(t,u(t) \bigr) \bigr\vert \,dt = & \int_{g(t,u(t))\geq 0}g^{+} \bigl(t,u(t) \bigr)\,dt- \int_{g(t,u(t))\leq 0}g^{-} \bigl(t,u(t) \bigr)\,dt \\ = &-2 \int_{g(t,u(t))\leq 0 }g^{-} \bigl(t,u(t) \bigr)\,dt+ \int^{\omega }_{0}f \bigl(t,u'(t) \bigr)\,dt \\ \leq &2a \int^{\omega }_{0} \bigl\vert u(t) \bigr\vert ^{p-1}\,dt+2b\omega +K\omega \\ \leq &2a\omega \Vert u\Vert _{\infty }^{p-1}+2b\omega +K \omega, \end{aligned}$$

where $g^{-}:=\min \{g(t,u),0\}$. Substituting (3.4) and (3.7) into (3.6), we have

$$\begin{aligned} \int^{\omega }_{0} \bigl\vert (Au)'(t) \bigr\vert ^{p}\,dt \leq & \bigl(1+\vert c\vert \bigr)\Vert u \Vert _{\infty } \bigl( 2a \omega \Vert u\Vert _{\infty }^{p-1}+2b \omega +2K\omega +\Vert e\Vert _{\infty } \omega \bigr) \\ = &2 \bigl(1+\vert c\vert \bigr)a\omega \Vert u\Vert _{\infty }^{p}+ \bigl(1+\vert c\vert \bigr)N_{1} \Vert u\Vert _{\infty } \\ \leq &2 \bigl(1+\vert c\vert \bigr)a\omega \biggl( D_{1}+ \frac{1}{2} \int^{\omega }_{0} \bigl\vert u'(t) \bigr\vert \,dt \biggr) ^{p} \\ &{}+ \bigl(1+\vert c\vert \bigr)N_{1} \biggl( D_{1}+\frac{1}{2} \int^{\omega }_{0} \bigl\vert u'(t) \bigr\vert \,dt \biggr) \\ = &\frac{(1+\vert c\vert )a\omega }{2^{p-1}} \biggl( 1+\frac{2D_{1}}{\int^{\omega }_{0}\vert u'(t)\vert \,dt} \biggr) ^{p} \biggl( \int^{\omega }_{0} \bigl\vert u'(t) \bigr\vert \,dt \biggr) ^{p} \\ &{}+ \frac{1}{2} \bigl(1+\vert c\vert \bigr)N_{1} \int^{\omega }_{0} \bigl\vert u'(t) \bigr\vert \,dt+ \bigl(1+\vert c\vert \bigr)N_{1}D _{1}, \end{aligned}$$

where $N_{1}:=2b\omega +2K\omega +\Vert e\Vert _{\infty }\omega $. For a given constant $\zeta >0$, which is only dependent on $k>0$, we have

$$(1+u)^{k}\leq 1+(1+k)u,\quad \mbox{for } u\in [0,\zeta ]. $$

Therefore, we have

3.8 $$\begin{aligned} \int^{\omega }_{0} \bigl\vert (Au)'(t) \bigr\vert ^{p}\,dt \leq & \frac{(1+\vert c\vert )a\omega }{2^{p-1}} \biggl( 1+ \frac{2D_{1}p}{\int^{\omega } _{0}\vert u'(t)\vert \,dt} \biggr) \biggl( \int^{\omega }_{0} \bigl\vert u'(t) \bigr\vert \,dt \biggr) ^{p} \\ &{}+ \frac{1}{2} \bigl(1+\vert c\vert \bigr)N_{1} \int^{\omega }_{0} \bigl\vert u'(t) \bigr\vert \,dt+ \bigl(1+\vert c\vert \bigr)N_{1}D _{1} \\ = &\frac{(1+\vert c\vert )a\omega }{2^{p-1}} \biggl( \int^{\omega }_{0} \bigl\vert u'(t) \bigr\vert \,dt \biggr) ^{p}+\frac{(1+\vert c\vert )a\omega D_{1}p}{2^{p-2}} \biggl( \int^{\omega }_{0} \bigl\vert u'(t) \bigr\vert \,dt \biggr) ^{p-1} \\ &{}+\frac{1}{2} \bigl(1+\vert c\vert \bigr)N_{1} \int^{\omega }_{0} \bigl\vert u'(t) \bigr\vert \,dt+ \bigl(1+\vert c\vert \bigr)N_{1}D _{1}. \end{aligned}$$

By application of Lemma 2.1, we have

3.9 $$\begin{aligned} \int^{\omega }_{0} \bigl\vert u'(t) \bigr\vert \,dt = & \int^{\omega }_{0} \bigl\vert \bigl(A^{-1}Au' \bigr) (t) \bigr\vert \,dt \\ \leq &\frac{\int^{\omega }_{0}\vert (Au)'(t)\vert \,dt}{\vert 1-\vert c\vert \vert } \\ \leq & \frac{\omega^{\frac{1}{q}} ( \int^{\omega }_{0}\vert (Au)'(t)\vert ^{p}\,dt) ^{\frac{1}{p}}}{\vert 1-\vert c\vert \vert }, \end{aligned}$$

since $(Au')(t)=(Au)'(t)$ and $\frac{1}{p}+\frac{1}{q}=1$. Apply the inequality

$$(a+b)^{k}\leq a^{k}+ b^{k},\quad \mbox{for } a, b>0, 0< k< 1. $$

Substituting (3.8) into (3.9), we have

$$\begin{aligned} \int^{\omega }_{0} \bigl\vert u'(t) \bigr\vert \,dt \leq & \frac{\omega^{\frac{1}{q}} ( \frac{(1+\vert c\vert )a \omega }{2^{p-1}} ) ^{\frac{1}{p}}\int^{\omega }_{0}\vert u'(t)\vert \,dt + \omega^{\frac{1}{q}} ( \frac{(1+\vert c\vert )a\omega D_{1}p}{2^{p-2}} ) ^{\frac{1}{p}} ( \int^{\omega }_{0}\vert u'(t)\vert \,dt) ^{\frac{p-1}{p}}}{\vert 1-\vert c\vert \vert } \\ &{}+\frac{\omega^{\frac{1}{q}} ( \frac{1}{2}(1+\vert c\vert )N_{1}\int^{ \omega }_{0}\vert u'(t)\vert \,dt) ^{\frac{1}{p}}+\omega^{\frac{1}{q}} ( (1+\vert c\vert )N _{1}D_{1} ) ^{\frac{1}{p}}}{\vert 1-\vert c\vert \vert }. \end{aligned}$$

Since $\frac{\omega (1+\vert c\vert )^{\frac{1}{p}}a^{\frac{1}{p}}}{\vert 1-\vert c\vert \vert }<2^{ \frac{p-1}{p}}$, it is easy to see that there exists a positive constant $M_{1}'$ such that

3.10 $$ \int^{\omega }_{0} \bigl\vert u'(t) \bigr\vert \,dt\leq M_{1}'. $$

From (3.4) and (3.10), we have

3.11 $$ \Vert u\Vert _{\infty }\leq D_{1}+ \frac{1}{2} \int^{\omega }_{0} \bigl\vert u'(t) \bigr\vert \,dt \leq D_{1}+\frac{1}{2}M_{1}':=M_{1}. $$

As $(Au)(0)=(Au)(\omega)$, there exists $t_{1}\in [0,\omega ]$ such that $(Au)'(t_{1})=0$, while $\phi_{p}(0)=0$, we have

3.12 $$\begin{aligned} \bigl\vert \phi_{p} \bigl((Au)'(t) \bigr) \bigr\vert = & \biggl\vert \int^{t}_{t_{1}} \bigl(\phi_{p} \bigl((Au)'(s) \bigr) \bigr)'\,ds \biggr\vert \\ \leq & \lambda \int^{\omega }_{0} \bigl\vert f \bigl(t,u'(t) \bigr) \bigr\vert \,dt+\lambda \int^{\omega }_{0} \bigl\vert g \bigl(t,u(t) \bigr) \bigr\vert \,dt+\lambda \int^{\omega }_{0} \bigl\vert e(t) \bigr\vert \,dt, \end{aligned}$$

where $t\in [t_{1},t_{1} +\omega ]$. In view of $(H_{1})$, (3.7) and (3.12), we have

3.13 $$\begin{aligned} \bigl\Vert \phi_{p}(Au)' \bigr\Vert _{\infty } = &\max_{t\in [0,\omega ]} \bigl\{ \bigl\vert \phi_{p} \bigl((Au)'(t) \bigr) \bigr\vert _{\infty } \bigr\} \\ = &\max_{t\in [t_{1},t_{1}+\omega ]} \biggl\{ \biggl\vert \int^{t}_{t_{1}} \bigl(\phi _{p} \bigl((Au)'(s) \bigr) \bigr)'\,ds \biggr\vert \biggr\} \\ \leq &\lambda \biggl( \int^{\omega }_{0} \bigl\vert f \big(t,u'(t) \bigr\vert \,dt+ \int^{\omega } _{0} \bigl\vert g \bigl(t,u(t) \bigr) \bigr\vert \,dt+ \int^{\omega }_{0} \bigl\vert e(t) \bigr\vert \,dt \biggr) \\ \leq &\lambda \bigl( K\omega +2a\omega \Vert u\Vert _{\infty }^{p-1}+2b \omega +K\omega +\Vert e\Vert _{\infty }\omega \bigr) \\ \leq &\lambda \bigl( 2a\omega M_{1}^{p-1}+2K\omega +2b\omega +\Vert e\Vert _{\infty }\omega \bigr):=\lambda M_{2}'. \end{aligned}$$

We claim that there exists a positive constant $M_{2}>M_{2}'+1$ such that, for all $t\in \mathbb{R}$,

3.14 $$ \bigl\Vert u' \bigr\Vert _{\infty }\leq M_{2}. $$

In fact, if $u'$ is not bounded, there exists a positive constant $M_{2}''$ such that $\Vert u'\Vert _{\infty }>M_{2}''$ for some $u'\in \mathbb{R}$. Therefore, we have

$$\begin{aligned} \bigl\Vert \phi_{p}(Au)' \bigr\Vert _{\infty } =& \bigl\Vert \phi_{p} \bigl(Au' \bigr) \bigr\Vert _{\infty }= \bigl\Vert Au' \bigr\Vert _{ \infty }^{p-1} \\ =& \bigl(1+\vert c\vert \bigr)^{p-1} \bigl\Vert u' \bigr\Vert _{\infty }^{p-1}\geq \bigl(1+\vert c\vert \bigr)^{p-1}M _{2}^{\prime\prime \, p-1}:=M_{2}^{*}. \end{aligned}$$

Then it is a contradiction. So (3.14) holds.

On the other hand, it follows by (2.1) that

3.15 $$ \bigl(\phi_{p}(Au)'(t) \bigr)'+\lambda f \bigl(t,u'(t) \bigr)+\lambda \bigl(g_{0} \bigl(u(t) \bigr)+g_{1} \bigl(t,u(t) \bigr) \bigr)= \lambda e(t). $$

Multiplying both sides of (3.15) by $u'(t)$ we get

3.16 $$\begin{aligned}& \bigl(\phi_{p}(Au)'(t) \bigr)'u'(t)+\lambda f \bigl(t,u'(t) \bigr)u'(t)+\lambda \bigl(g_{0} \bigl(u(t) \bigr)+g _{1} \bigl(t,u(t) \bigr) \bigr)u'(t) \\& \quad =\lambda e(t)u'(t). \end{aligned}$$

Let $\tau \in [0,\omega ]$ be as in (3.3), for any $\tau \leq t\leq \omega$, we integrate (3.16) on $[\tau,t]$ and get

3.17 $$\begin{aligned} \lambda \int^{u(t)}_{u(\tau)}g_{0}(v)\,dv = &\lambda \int^{t}_{\tau }g _{0} \bigl(u(s) \bigr)u'(s)\,ds \\ = &- \int^{t}_{\tau } \bigl(\phi_{p}(Au)'(s) \bigr)'u'(s)\,ds-\lambda \int^{t}_{ \tau }f \bigl(s,u'(s) \bigr)u'(s)\,ds \\ &{}-\lambda \int^{t}_{\tau }g_{1} \bigl(s,u(s) \bigr)u'(s)\,ds+\lambda \int^{t}_{ \tau }e(s)u'(s)\,ds. \end{aligned}$$

By (3.7), (3.11) and (3.14), we have

$$\begin{aligned} &\biggl\vert \int^{t}_{\tau } \bigl(\phi_{p}(Au)'(s) \bigr)'u'(s)\,ds \biggr\vert \\ &\quad \leq \int^{T} _{0} \bigl\vert \bigl( \phi_{p}(Au)'(s) \bigr)' \bigr\vert \bigl\vert u'(s) \bigr\vert \,ds \\ &\quad \leq \lambda \bigl\Vert u' \bigr\Vert _{\infty } \biggl( \int^{\omega }_{0}\big\vert f \bigl(t,u'(t) \bigr)\,dt+ \int^{\omega }_{0} \bigl\vert g \bigl(t,u(t) \bigr) \bigr\vert \,dt+ \int^{\omega }_{0} \bigl\vert e(t) \bigr\vert \,dt \biggr) \\ &\quad \leq \lambda M_{2} \bigl( K\omega +2a\omega \vert u\Vert _{\infty }^{p-1}+2b \omega +K\omega +\Vert e\Vert _{\infty }\omega \bigr) \\ &\quad \leq\lambda M_{2} \bigl( 2K\omega +2a\omega M_{1}^{p-1}+2b \omega + \Vert e\Vert _{\infty }\omega \bigr). \end{aligned}$$

Moreover, from $(H_{1})$ and (3.14)

$$\begin{aligned} & \biggl\vert \int^{t}_{\tau }f \bigl(s,u'(s) \bigr)u'(s)\,ds \biggr\vert \leq \int^{T}_{0} \bigl\vert f \bigl(s,u'(s) \bigr) \bigr\vert \bigl\vert u'(s) \bigr\vert \,ds \leq KM_{2} \omega, \\ & \biggl\vert \int^{t}_{\tau }g_{1} \bigl(s,u(s) \bigr)u'(s)\,ds \biggr\vert \leq \int^{T}_{0} \bigl\vert g_{1} \bigl(s,u(s) \bigr) \bigr\vert \bigl\vert u'(s) \bigr\vert \,ds \leq M_{2} \vert g_{M_{1}}\vert \sqrt{\omega }, \end{aligned}$$

where $g_{M_{1}}=\max_{0\leq u\leq M_{1}}\vert g_{1}(t,u)\vert \in L^{2}(0, \omega)$.

$$\biggl\vert \int^{t}_{\tau }e(s)u'(s)\,ds \biggr\vert \leq \int^{\omega }_{0} \bigl\vert e(s) \bigr\vert \bigl\vert u'(s) \bigr\vert \,ds \leq \Vert e\Vert _{\infty } \omega M_{2}. $$

With these inequalities we can derive from (3.17) that

$$\biggl\vert \int^{u(t)}_{u(\tau)}g_{0}(v)\,dv \biggr\vert \leq M_{2} \bigl(3K\omega +2a \omega M_{1}^{p-1}+2b \omega +2\Vert e\Vert _{\infty }\omega +\vert g_{M_{1}}\vert \sqrt{ \omega } \bigr). $$

In view of (1.6), we know there exists $M_{3}>0$ such that

3.18 $$ u(t)\geq M_{3},\quad \forall t\in [\tau,\omega ]. $$

The case $t\in [0,\tau ]$ can be treated similarly.

Having in mind (3.11), (3.14) and (3.18), we define

$$\Omega = \bigl\{ u\in X:E_{1}< u(t)< E_{2} \mbox{ and } \bigl\vert u'(t) \bigr\vert < E_{3}\ \forall t\in \mathbb{R} \bigr\} , $$

where $0< E_{1}< \min \{D_{2},M_{3}\}$, $E_{2}>\max \{M_{1}, D_{1}\} $ and $E_{3}>M_{2}$. We know that (2.1) has no solution on *∂*Ω as $\lambda \in (0,1)$ and when $u(t)\in \partial \Omega \cap \mathbb{R}$, $u(t)=E_{2}$ or $u(t)=E_{1}$, from (3.4), we know that $E_{2}+1>D_{1}$; therefore, from $(H_{2})$ we see that

$$\frac{1}{\omega } \int^{\omega }_{0}g(t,E_{2})\,dt< 0 $$

and

$$\frac{1}{\omega } \int^{\omega }_{0}g(t,E_{1})\,dt>0. $$

So condition (ii) is also satisfied. Set

$$H(u,\mu)=\mu u+(1-\mu)\frac{1}{\omega } \int^{\omega }_{0}g(t,u)\,dt, $$

where $x\in \partial \Omega \cap \mathbb{R}$, $\mu \in [0,1]$, we have

$$uH(u,\mu)=\mu u^{2}+(1-\mu)\frac{u}{\omega } \int^{\omega }_{0}g(t,u)\,dt \neq 0, $$

and thus $H(u,\mu)$ is a homotopic transformation and

$$\begin{aligned} \deg \{F,\Omega \cap \mathbb{R},0\} &=\deg \biggl\{ \frac{1}{\omega } \int^{\omega }_{0}g(t,u)\,dt,\Omega \cap \mathbb{R},0 \biggr\} \\ &=\deg \{u,\Omega \cap \mathbb{R},0\}\neq 0. \end{aligned}$$

So condition (iii) is satisfied. In view of Lemma 2.1, there exists a solution with period *ω*. □

## Example

### Example 4.1

Consider the following *p*-Laplacian neutral Rayleigh equation with singularity:

4.1 $$\begin{aligned}& \biggl( \phi_{p} \biggl( u(t)- \frac{1}{4}u ( t-\delta) \biggr) ' \biggr) '- \cos^{2}(2t)\sin u'(t)-\frac{1}{3\pi^{4}}( \sin 4t+2)u^{3}(t)+\frac{1}{u ^{\mu }} \\& \quad =\sin^{2}(2t), \end{aligned}$$

where $\mu \geq 1$ and $p=4$, *δ* is a constant and $0\leq \delta <\omega $.

It is clear that $\omega =\frac{\pi }{2}$, $c=\frac{1}{4}$, $e(t)=\sin ^{2}(2t)$, $f(t,v)=-\cos^{2}(2t)\sin v$, $g(t,u)=-\frac{1}{3\pi^{3}}( \sin 4t+2)u^{4}(t)+\frac{1}{u^{\mu }(t)}$. Choose $K=1$, $D_{1}=2$, $D_{2}=1$, $a=\frac{1}{\pi^{4}}$, it is obvious that $(H_{1})$, $(H_{2})$ and $(H_{3})$ hold. Next, we consider

$$\begin{aligned} & \frac{\omega (1+\vert c\vert )^{\frac{1}{p}}a^{\frac{1}{p}}}{2^{\frac{p-1}{p}}\vert 1-\vert c\vert \vert } \\ &\quad =\frac{\frac{\pi }{2}(1+\frac{1}{4})^{\frac{1}{4}} ( \frac{1}{\pi ^{4}} ) ^{\frac{1}{4}}}{2^{\frac{3}{4}} ( 1-\frac{1}{4} ) } \\ &\quad \approx\frac{1.057}{1.783}< 1. \end{aligned}$$

Therefore, by Theorem 3.1, (4.1) has at least one nonconstant $\frac{\pi }{2}$-periodic solution.

## Conclusions

In this article we introduce the existence of a periodic solution for a *p*-Laplacian neutral Rayleigh equation with singularity of attractive type. Due to the attractive condition being in contradiction with the repulsive condition, the methods of [12, 15, 16] are no long applicable to the proof of a periodic solution for equation (1.5) with singularity of attractive singularity. In this paper, we give attractive conditions (1.6) and $(H_{3})$, and we see the existence of a periodic solution for (1.5) by applications of the extension of Mawhin’s continuation theorem [17]. Moreover, in view of the mathematical points, the results satisfying the conditions of an attractive singularity are valuable to understand the periodic solution for Rayleigh equations.
